# The remarkable scorpion diversity in the Ecuadorian Andes and description of a new species of *Tityus* C. L. Koch, 1836 (Scorpiones, Buthidae)

**DOI:** 10.3897/zookeys.307.5334

**Published:** 2013-06-05

**Authors:** Wilson R. Lourenço, Eric Ythier

**Affiliations:** 1 Muséum national d’Histoire naturelle, Département Systématique et Evolution, UMR7205, CP 053, 57 rue Cuvier, 75005 Paris, France; 2 SynTech Research, 613 route du Bois de Loyse, 71570 La Chapelle de Guinchay, France

**Keywords:** Scorpiones, Buthidae, *Tityus*, Ecuador, New Species, diversity

## Abstract

A new species of *Tityus*, subgenus *Atreus* (Scorpiones: Buthidae) is described from the Province of Pichincha in the Ecuadorian Andes. Ecuadorian scorpion fauna remains one of the less well studied among those of South America. Nevertheless, some comments are addressed about its remarkable diversity and high level of endemic elements.

## Introduction

The scorpion fauna inhabiting the regions between Southern Colombia and the Ecuadorian Andes has attracted the attention of experts since the middle of the 19^th^ century (e.g. Gervais 1844; Thorell 1876; Pocock 1893, 1898; Borelli 1899; Kraepelin 1912a, b; Mello-Leitão 1945). Only since the 1980s has this fauna been more frequently studied and several new species have been described (Lourenço 1984, 1988, 1991, 1992, 1994a, 1995, 1997, 1999, 2000; Botero-Trujillo 2008, 2009; Botero-Trujillo and Francke 2009).

Although studies by Lourenço (1988, 1995) represented the first attempt to produce a synthesis of the Ecuadorian scorpion fauna, these contributions probably only represented a small part of the fauna actually present in Ecuador. This conjecture is supported by the descriptions of new species from different regions of the country (e. g. Lourenço 2005, 2007). In the winter (southern hemisphere) of 2012, scorpions were collected from mountains within the Ecuadorian Andes. Two specimens (1 male, 1 female) were discovered to be a new species belonging to the genus *Tityus*. Herein, we describe this new species and comment on the scorpion fauna found within the Ecuadorian region, and suggest several hypotheses regarding mechanisms that generated this remarkable diversity.

## Methods

Illustrations and measurements were made with the aid of a Wild M5 stereo-microscope with a drawing tube (camera lucida) and an ocular micrometer. Measurements follow Stahnke (1970) and are given in mm. Trichobothrial notations follow Vachon (1974) while morphological terminology mostly follows Hjelle (1990).

## Taxonomic treatment

### Family Buthidae C. L. Koch, 1837 Genus *Tityus* C. L. Koch, 1836 Subgenus *Atreus* Gervais, 1843

#### Tityus (Atreus) crassicauda sp. n.

http://species-id.net/wiki/Tityus_crassicauda

urn:lsid:zoobank.org:act:2F53526B-67DB-419D-8F64-650AF221B19E

Figs 1 –18

##### Material examined.

Ecuador, Pichincha Province, Tandayapa, 2200 m, VIII/2012 (G. Onore & I. Tapia leg.). Inside of Lauraceae rotten logs.

Male holotype and female paratype. Deposited in the Muséum national d’Histoire naturelle, Paris.

##### Etymology.

Specific name refers to the strongly enlarged posterior metasomal segments.

##### Diagnosis.

Scorpions of medium size in relation to other species within the genus. Total length in male and female, 51.9 and 50.5 mm, respectively. General coloration reddish-brown with darker blackish zones; carapace and tergites with three longitudinal brownish stripes, separated by yellow zones. Metasomal segments flattened, comparatively to other species of the group; segments IV and V very strongly enlarged, especially in the male holotype. Dorsal carinae of metasomal II to IV terminating distally with two very strong spinoid granules, more distinct on the female paratype. Pectines small with 15–15 teeth on male and 15–14 on female; basal piece of the middle lamella strongly dilated on female, and oval in shape. Cutting edges of fixed and movable fingers of pedipalp chela with 12–13 and 12–14 rows of granules on the male and female, respectively.

Relationships. The new species is clearly allied to *Tityus forcipula* (Gervais, 1843) and other associated species such as *Tityus spinatus* Pocock, 1898 and *Tityus cuellari* Lourenço, 1994, species also known from the Colombian and Ecuadorian Andes and, previously defined as part of the *Tityus forcipula* complex (see Lourenço 1984 for details). It can, however, be distinguished from all these species and in particular from *Tityus forcipula* by a number of features: (i) a distinct pattern of coloration and pigmentation, with brownish longitudinal stripes on the carapace and tergites, which are absent from the other species, (ii) sternites III to VII with a very strongly marked granulation but a moderately marked setation; granulations are less marked on the other species, (iii) metasomal segments strongly flattened and very strongly enlarged, especially in the male holotype; less enlarged in the other species, (iv) tergites with only moderately marked granulation, (v) pedipalp segments and chela are smooth to lustrous; somewhat granular in the other species.

Description based on male holotype and female paratype (measurements in Table 1).

Coloration. Reddish-brown with darker, blackish areas. Prosoma: carapace reddish-brown, with yellowish zones between posterior median carinae. Mesosoma: reddish-brown with three brown to blackish longitudinal stripes extending from the posterior zone of the carapace and over tergites I to VII. Metasomal segments I to III reddish-brown, with dark to blackish carinae; IV and V reddish-brown dorsally, blackish laterally and ventrally. Vesicle: brownish-black; aculeus yellowish. Venter is reddish-brown, with some yellowish zones. Chelicerae yellowish-brown, with a very dark thread of variegated spots; fingers dark; teeth reddish. Pedipalps: reddish; fingers blackish, with extremities yellowish. Legs reddish-yellow to reddish-brown, with some diffuse and discrete fuscous spots; tarsi yellowish.

**Table 1.** Measurements (in mm) of male holotype and female paratype of *Tityus crassicauda* sp. n., and of male and female of *Tityus forcipula* from Pichincha. <br/>

	***Tityus crassicauda* sp. n.**		***Tityus forcipula***	
	♂	♀	♂	♀
Total length (including telson)	51.9	50.5	58.4	57.8
Carapace				
length	5.7	5.7	6.4	6.3
anterior width	3.9	3.9	4.5	4.4
posterior width	6.5	6.9	7.4	7.8
Mesosoma length:	13.3	14.9	13.6	17.8
Metasomal segment I:				
length	4.1	3.7	4.3	4.4
width	3.7	3.5	4.2	3.7
Metasomal segment II:				
length	4.9	4.2	5.5	4.6
width	4.0	3.3	4.3	3.8
Metasomal segment III:				
length	5.3	4.6	6.2	5.4
width	4.3	3.5	4.8	3.9
Metasomal segment IV:				
length	5.9	5.2	7.2	5.9
width	5.4	4.0	5.3	4.5
Metasomal segment V:				
length	6.5	6.1	8.3	6.8
width	5.8	4.3	5.3	4.8
depth	3.2	2.6	4.3	3.6
Telson length:	6.2	6.1	6.9	6.6
Vesicle:				
width	3.0	2.6	3.3	3.2
depth	2.5	2.3	2.7	2.4
Pedipalp Femur:				
length	6.0	5.5	6.8	6.5
width	1.7	1.8	2.0	2.0
Pedipalp Patella:				
length	6.8	6.1	7.7	7.1
width	2.5	2.2	2.8	2.4
Pedipalp Chela:				
length	11.6	10.2	13.2	12.1
width	3.4	2.2	4.1	2.5
depth	3.5	2.0	3.9	2.6
Movable finger:				
length	7.6	7.1	8.2	8.2

Morphology. Carapace moderately to strongly granular; anterior margin with a moderately marked concavity. Anterior median superciliary and posterior median carinae moderate to strong. Furrows moderately to strongly deep. Median ocular tubercle distinctly anterior to the centre of carapace. Eyes separated by a little more than one ocular diameter. Three pairs of lateral eyes. Sternum triangular. Mesosomal tergites moderately granular. Median carina strong on all tergites. Tergite VII pentacarinate. Venter: genital operculum divided longitudinally, forming two semi-oval plates. Pectines small; pectinal tooth count 15–15 in male holotype and 15–14 in female paratype; basal piece of the middle lamellae of the female pectines oval shaped and strongly dilated. Sternites very strongly granular, in particular on male holotype; spiracles moderately elongated; VII with four moderately to strongly marked carinae. Metasomal segments strongly flattened dorsally; IV and V very strongly enlarged, especially in the male holotype; segments I and II with ten carinae; III and IV with eight carinae, crenulate; V with five carinae. Dorsal carinae on segments II to IV with two very strong spinoid granules, distally. Lateral inframedian carinae on segment I complete, crenulate; on II almost complete; absent from III and IV. Ventrolateral carinae strong, crenulated; ventral submedian carinae strongly crenulate. Intercarinal spaces moderately granular. Segment V with weak dorsolateral carinae; ventrolateral and ventromedian carinae strong, crenulate. Lateral intercarinal spaces moderately to strongly granular. Telson moderately granular, with a long, strongly curved aculeus. Dorsal surface smooth; ventral surface weakly granular; subaculear tooth strong and spino-rhomboidal in shape. Cheliceral dentition characteristic of the family Buthidae (Vachon, 1963); movable finger with two basal teeth well distinct; ventral aspect of both fingers and manus with long, dense setae. Pedipalps: femur pentacarinate; patella with seven carinae; chela with nine carinae, more distinct on female paratype; all faces with only a few granules; smooth. Fixed and movable fingers of chela with respectively 12 and13 oblique rows of granules on the male holotype and 12–14 on the female paratype. Trichobothriotaxy; orthobothriotaxy A-α (alpha) (Vachon 1974, 1975). Legs: tarsus with two series of 6–7 setae on ventral surface.

**Figures 1–2.**
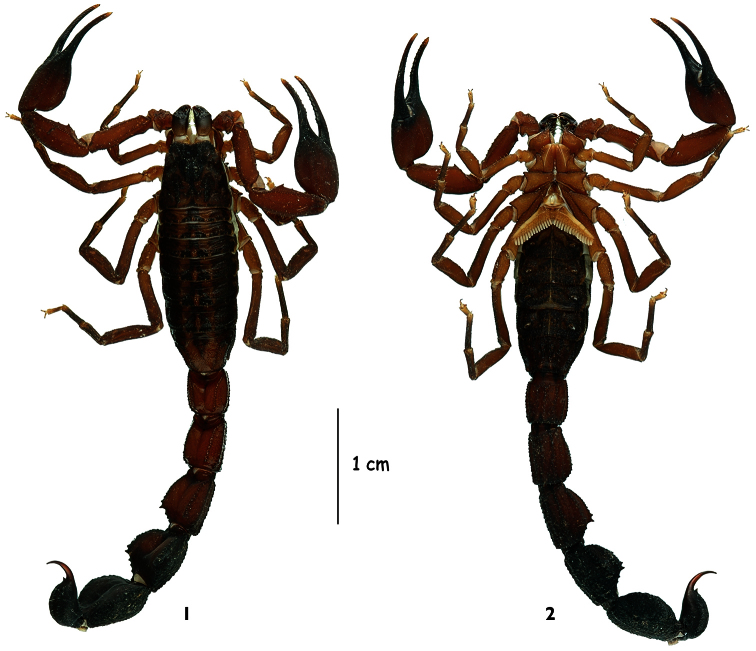
*Tityus (Atreus) crassicauda* sp. n., male holotype, dorsal and ventral aspects.

**Figures 3–4.**
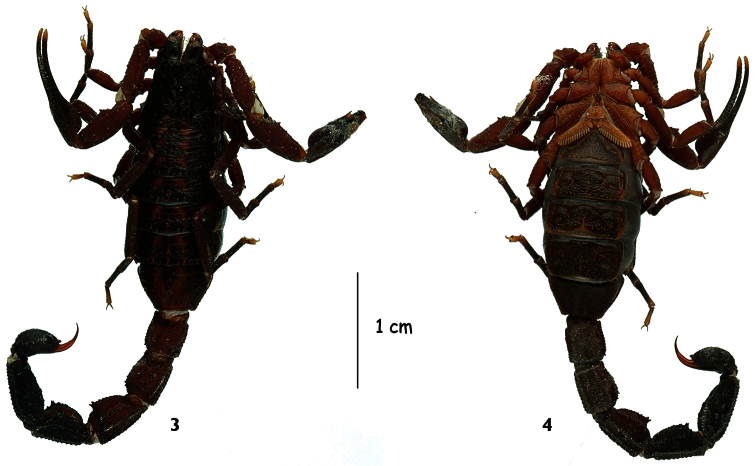
*Tityus (Atreus) crassicauda* sp. n., female paratype, dorsal and ventral aspects.

**Figures 5–9.**
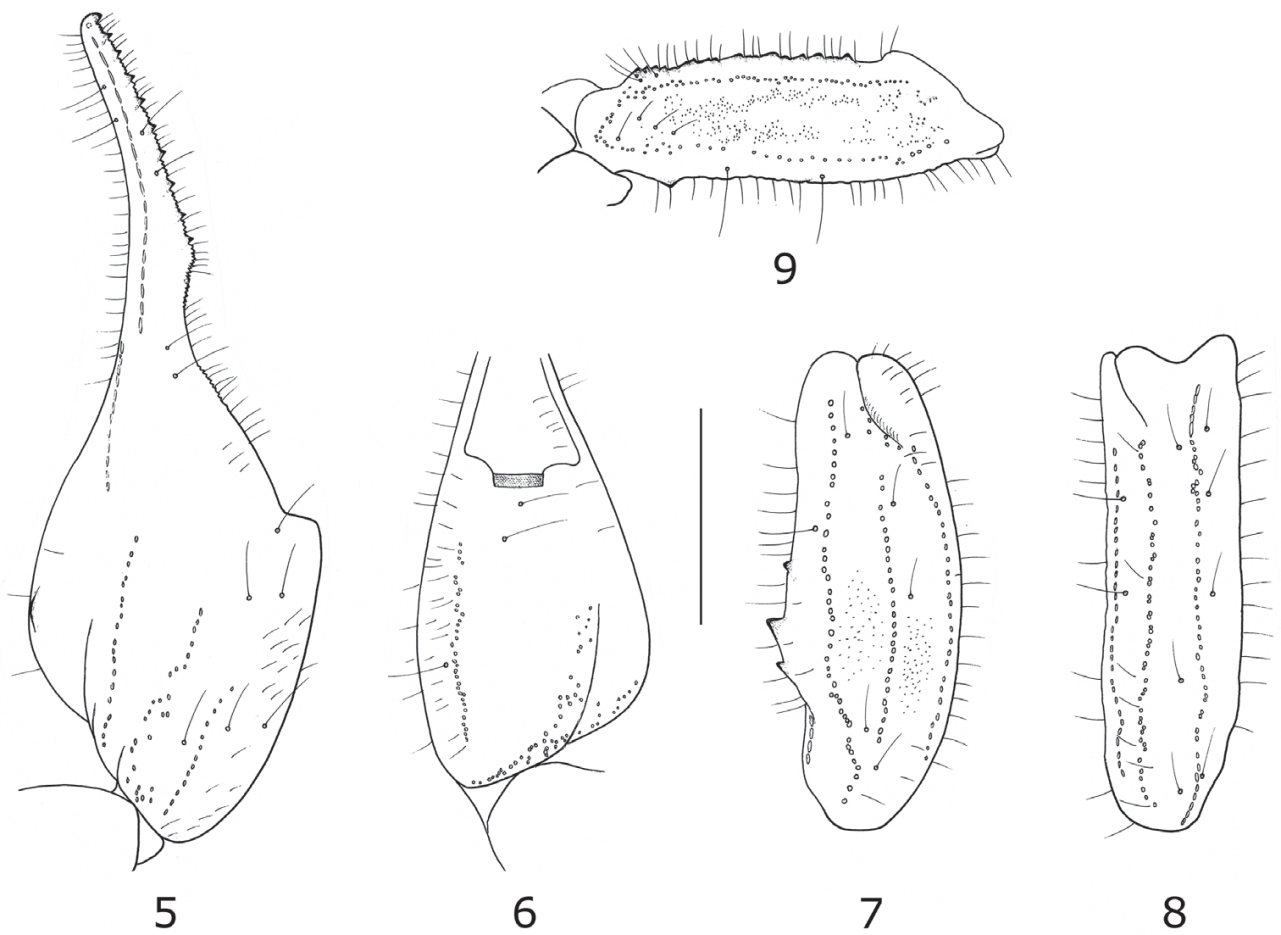
*Tityus (Atreus) crassicauda* sp. n. Trichobothrial pattern of male holotype **5–6** Chela dorso-external and ventral aspects **7–8** Patella, dorsal and external aspects **9** Femur, dorsal aspect (scale bar = 3 mm).

**Figures 10–12.**
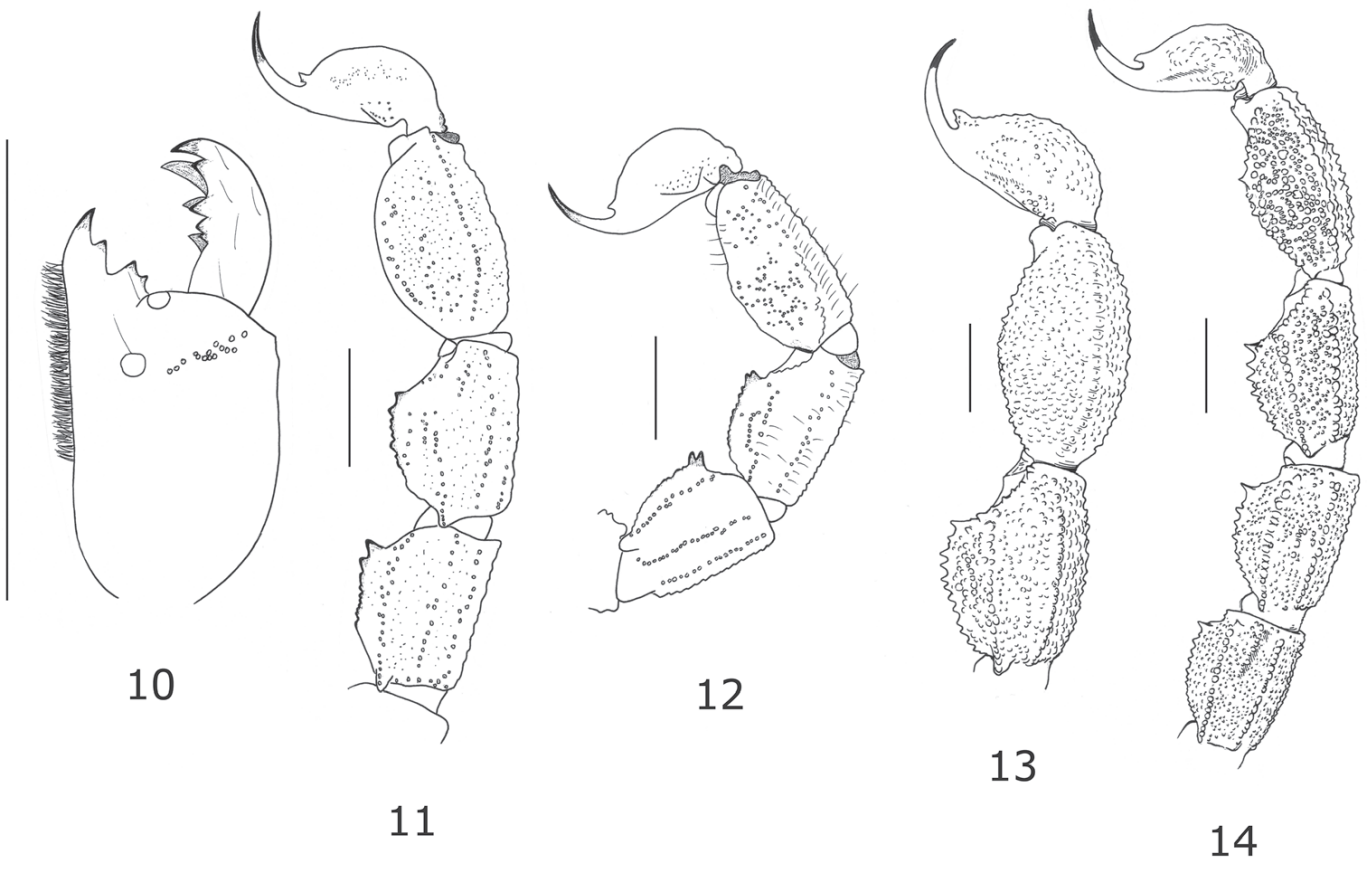
*Tityus (Atreus) crassicauda* sp. n. **10** Right chelicera, dorsal aspect (male holotype) **11–12** Metasomal segments III-V and telson, lateral aspect (male holotype and female paratype) **13** Segments IV, V and telson for *Tityus (Atreus) forcipula*, male holotype **14** Segments II to V and telson for *Tityus (Atreus) spinatus*, female holotype (scale bars = 3 mm).

**Figure 15.**
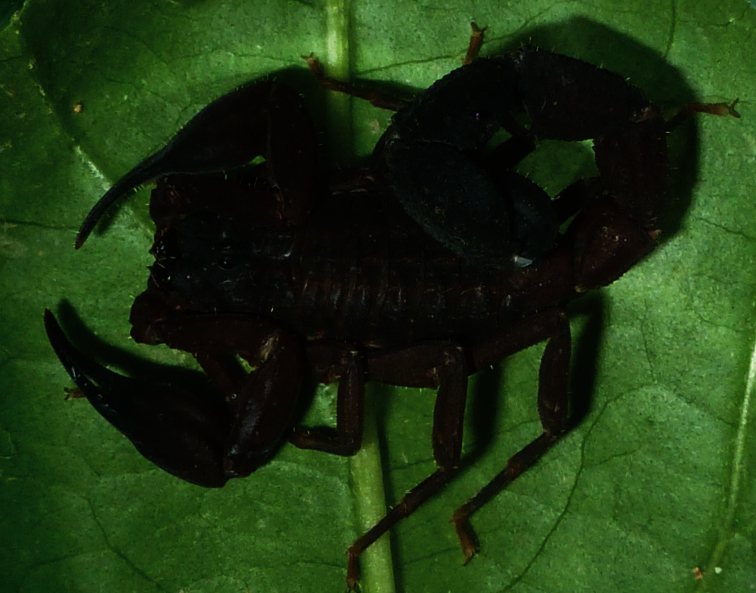
*Tityus (Atreus) crassicauda* sp. n. Male holotype alive in the field.

**Figures 16–17.**
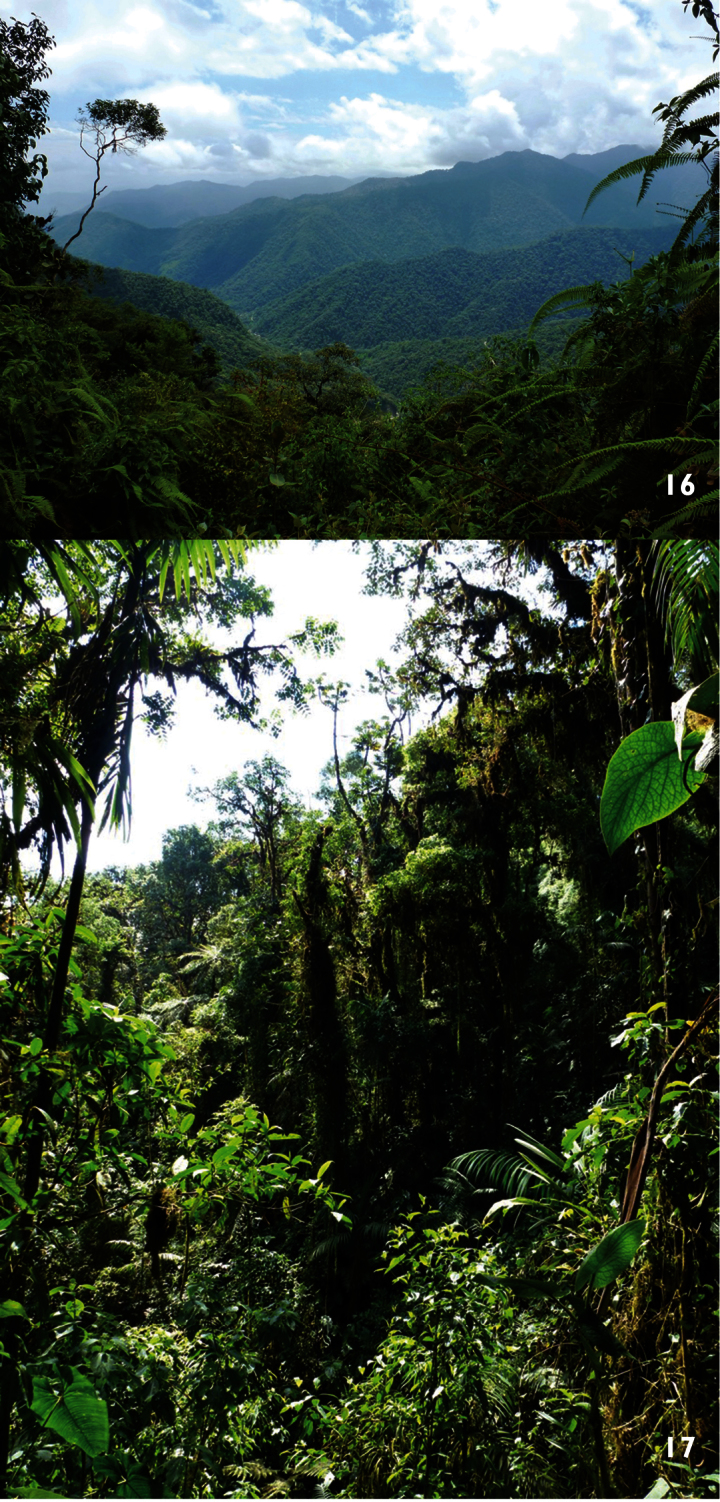
The natural habitat of *Tityus (Atreus) crassicauda* sp. n. **16** General view of the Andean Mountains **17** Detail of the vegetation cover.

##### Habitat.

The new species inhabits the tropical forests of the Ecuadorian Andean Mountains, in the Pichincha Province. The specimens were collected at an altitude of 2200 m, inside of rotten Lauraceae logs.

**Figure 18.**
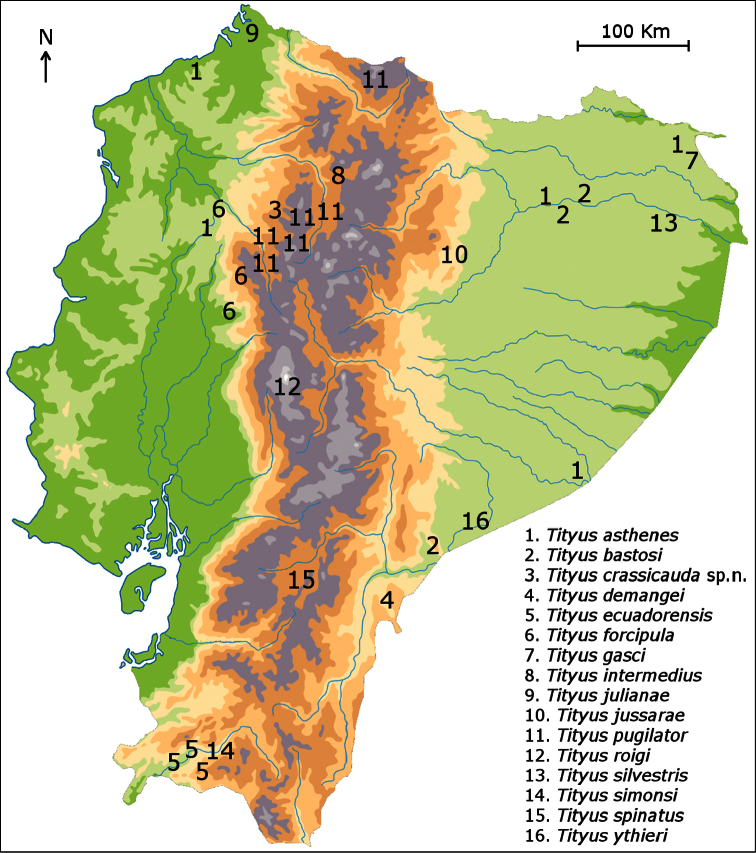
Map of Ecuador showing the distribution of the known species of *Tityus*.

## The remarkable diversity of the Ecuadorian scorpion fauna

As already outlined by Lourenço (1994b), many if not most authors working on the floras and faunas of the Neotropical region seem to agree that the possible ‘epicentre’ of global diversity occurs in the tropical and subtropical Andean region (the upper Amazon, North of Peru, and most of Ecuador and Southern Colombia). This hypothesis is supported by studies conducted on various taxa (plants, vertebrates and butterflies (Gentry 1988, 1992)). In the same line of thinking, Lourenço (1994b) suggested that one of the areas exhibiting the highest alpha-diversity for scorpions in the world ranged from Southern Colombia, Ecuador, the Northeast region of Peru, and the Upper Amazon region of Brazil. This region has been already the subject of other previous research programs, namely the ‘Transecto Ecuadorial Pacifico Amazonico’, as defined by Van Velzen (1992). Despite studies such as these, there still remain important gaps in the knowledge of scorpion communities within the Andean region. By expanding the area studied eastward, it would allow for a more accurate survey of Colombia, Peru and Brazil.

The number of endemic taxa in this region is very high - more than 80% - and is comparable to the number of endemic taxa found in other previously studied regions such as Baja California (about 75%) (Williams 1980; Lourenço 1994b). However, desert areas such as Baja California are much better studied than tropical areas. Consequently, a substantial increase in the total number of species in North American deserts is unlikely. The use of ultra-violet light in the detection of scorpions in open areas such as deserts has been intense for many decades. On the other hand, inventory work in tropical forests is still fairly new and UV light is not as efficient in this type of habitat, mainly because of the landscape vegetation. Moreover, for several sections of the tropical forests such as the canopy, knowledge of the scorpion fauna is virtually non-existent (Lourenço and Pézier 2002). Consequently, the effective number of species in rainforests may be much greater than what is presently estimated; see Lourenço (1994b) for comparative numbers.

The scorpion fauna of the Ecuadorian Andes is characterized by an outstanding concentration of species belonging to genera such as *Tityus* and *Teuthraustes* Simon, 1878 (Chactidae). Within each genus, the species show to be closely related. The speciation pattern of the scorpions corresponds to the explosive model proposed by Gentry (1992) for plants of the genus *Gasteranthus* which have a similar range of distribution in this same area. The proposed mechanism operating process is associated with some type of genetic transilience associated with genetic drift in small founder populations, a process also postulated for Hawaiian *Drosophila* (Gentry 1992).

## Acknowledgements

We are most grateful to Michael M. Webber, University of Nevada, Las Vegas for her comments and review of the manuscript and to two anonymous referees for useful comments and suggestions; to Elise-Anne Leguin, MNHN, Paris for the preparation of some photos and to Peter Schwendinger of the Geneva Museum for the loan of specimens of *Tityus forcipula*. We also want to express our sincere gratitude to Prof. G. Onore for providing the collected specimens.

## Supplementary Material

XML Treatment for Tityus (Atreus) crassicauda
